# An Evaluation of Practitioner Competency and Practices in Diagnosing and Managing Keratoconus in Malawi: A Cross-Sectional Analysis

**DOI:** 10.3390/vision10040079

**Published:** 2026-10-10

**Authors:** Leaveson Thom, Vanessa R. Moodley

**Affiliations:** 1 Discipline of Optometry, University of KwaZulu Natal, Durban 4000, South Africa; moodleyvr@ukzn.ac.za; 2 Keratoconus Foundation, Durban 4015, South Africa

**Keywords:** keratoconus, clinical competency, eye care practitioners, service delivery, contact lenses, Malawi

## Abstract

This study assessed eye care practitioners’ competencies, clinical practices, and barriers related to KC diagnosis and management in Malawi. A national cross-sectional study was conducted among eye care practitioners in Malawi. Data were collected using a structured questionnaire assessing demographics, self-reported competency in KC diagnosis and management, clinical practices, service availability, and barriers to care. Data analysis was conducted using Stata version 15 and R version 4.5.1. A total of 110 practitioners participated (response rate: 58.5%). The mean age was 35.4 ± 7.4 years, and optometrists constituted 60.9% of respondents. Diagnostic competency scores were consistently higher than management competency scores across knowledge, skills, and experience domains. Higher educational attainment was independently associated with diagnostic competency, with undergraduate (aOR = 3.3; 95% CI: 1.13–9.63) and postgraduate practitioners (aOR = 6.6; 95% CI: 1.91–22.62) demonstrating greater odds of competency than diploma holders. Equipment shortages were significantly associated with facility type (*p* = 0.035), while other barriers showed no significant variation across regions or levels of care. Eye care practitioners in Malawi reported moderate competency in KC diagnosis and management, with stronger confidence in diagnosis than management. Strengthening practitioner training, improving access to diagnostic technologies, and enhancing affordability of contact lens services may improve KC care delivery and visual outcomes.

## 1. Introduction

The presentation, clinical characteristics, and refractive effects of keratoconus (KC) have been described by European oculists from as early as the 18th and 19th centuries [1]. Presently, KC is defined as a bilateral and asymmetric ocular disease which results in progressive thinning and steepening of the cornea, leading to irregular astigmatism, visual impairment and reduced quality of life [2,3]. The exact causes of KC are still being investigated but it is believed to involve a genetic predisposition and environmental factors, like eye rubbing and nocturnal ocular compression [4,5,6]. Its prevalence varies across the globe, reportedly due to the differences in its definition and diagnostic approach, as well as the ethnicity, age, geographical factors, expertise, and cohort of the subjects, among other factors [5,6,7,8,9,10,11]. Diagnostic capabilities have improved with the advent of diagnostic tools such as corneal topography, and more recently corneal tomography, resulting in identification of this corneal ectasia at a much earlier stage than what was previously possible [11,12]. Consequently, the previously established prevalence rate of approximately 1/2000 among the general population has been challenged with much higher prevalence rates found in many parts of the world [8,9,13,14,15].

Effective KC management relies heavily on timely diagnosis, appropriate clinical decision making and coordinated, multidisciplinary care across different levels of the eye care system. Practitioner knowledge and clinical competence play critical roles in preventing avoidable vision loss associated with this condition. Further, early detection is paramount to enable intervention strategies that delay progression and also prevent iatrogenic ectasia in patients with subclinical forms of keratoconus who undergo collagen cross linking (CXL) and refractive surgery [16,17].

In many low- and middle-income countries (LMICs), including Malawi, keratoconus remains under detected, often misclassified as a refractive error. Despite the existence of a multidisciplinary, three-tier eye care service delivery system [18], there are no nationally established guidelines or clinical algorithms for keratoconus diagnosis and management. A key element of comprehensive disease management is the clinical competence of the team at the respective facility. Assessing the self-reported knowledge and skills of eye care practitioners provides valuable insight into the existing strengths and gaps in keratoconus management, guiding the development of targeted capacity-building interventions if necessary. While similar studies have been conducted in other African settings [19], there is a paucity of evidence from Malawi. This study, therefore, aims to evaluate the self-reported knowledge of eye care practitioners regarding the diagnosis and management of KC across public, non-governmental and private health facilities in Malawi. The aim of acquiring human resource capacity data is to inform policy, training programmes and the development of context appropriate clinical guidelines.

## 2. Methods

### 2.1. Study Design

This study employed a quantitative, cross-sectional design conducted across three regions of Malawi, the Northern, Central and Southern Regions.

### 2.2. Study Setting

The study was conducted in public, non-governmental (mission) and private facilities across Malawi. Currently, there are about 30 public hospitals with eye care facilities. Of these hospitals, four of them are central hospitals located in three administrative regions across Malawi.

### 2.3. Study Population

This study was conducted amongst all eye care practitioners registered under the medical council of Malawi. It only targeted those actively practicing in Malawi from March to December 2025.

### 2.4. Sample Size

This study employed a total population sampling (census) approach, whereby all eye care practitioners actively registered with the Medical Council of Malawi and practicing in public, mission/non-governmental, and private facilities between March and December 2025 were invited to participate. According to the 2025 Medical Council register, 188 eligible practitioners comprised the national eye care workforce during the study period. Of these, 110 practitioners completed the survey, yielding a response rate of 58.5%. Participants represented all three geographical regions of Malawi and all levels of eye care delivery (primary, secondary, and tertiary), providing broad national coverage of the practicing eye care workforce.

### 2.5. Data Collection Tools and Procedures

A self-administered, structured questionnaire was developed from the existing literature, validated, and piloted before being used for data collection.

The questionnaire consisted of closed-ended questions on eye care practitioner’s demographic details, education background, work experience, keratoconus knowledge and skills, equipment availability, and the diagnosis and management practices applied. Further, barriers to the diagnosis and management of keratoconus were also explored. The level of knowledge and skills were measured on a five-point Likert scale ranging from poor = 1, fair = 2, average = 3, good = 4 to excellent = 5. Descriptors were then used as follows; **Poor (1):** No or very limited knowledge, skills or clinical experience; unable to independently diagnose or manage keratoconus. **Fair (2):** Basic theoretical understanding but requires substantial supervision for clinical application. **Average (3):** Moderate knowledge and skills; able to manage uncomplicated cases with occasional guidance. **Good (4):** Sound theoretical knowledge and practical skills; able to independently diagnose and manage most keratoconus cases within the practitioner’s scope of practice. **Excellent (5):** Advanced knowledge, extensive clinical experience and confidence in diagnosing and managing keratoconus, including complex cases appropriate to the practitioner’s professional scope. Thereafter, total summations of all these five descriptors were calculated, and practitioners whose total summation was equal to or above the median score (50% more) were considered knowledgeable.

The questionnaire was administered physically and electronically through emails and Google forms, as well as using social media platforms like WhatsApp, Snapchat and Telegram. The data was then captured in an excel sheet before being entered into Stata version 15 and R version 4.5.1 for analysis.

### 2.6. Ethical Considerations

Ethical clearance to conduct this study was obtained from UKZN’s Humanities and Social Sciences Research Ethics Committee (HSSREC/00008212/2025) and Malawi’s National Committee on Research in the Social Sciences and Humanities (NO. P.10/24/926). All the procedures adhered to the ethical standards and guidelines of the Helsinki Declaration involving human subjects. Furthermore, gatekeeper letters were obtained from all the targeted facilities before the commencement of data collection.

### 2.7. Statistical Analysis

Data analysis was conducted using Stata version 15 and R version 4.5.1. New categorical and composite variables were developed to support analysis. Continuous variables were recoded into meaningful categories: Years of Experience was grouped as “<5 years,” “5–9 years,” and “≥10 years,” while Age was categorised as “<30 years,” “30–39 years,” and “≥40 years.” Composite competency scores for diagnosis and management were generated to calculate the mean scores of practitioners’ self-rated knowledge, skill and experience. These continuous composite scores were then dichotomised into binary outcomes (“below average” vs. “above average”) for inferential analyses assessing predictors of practitioner competency. Descriptive statistics were used to summarise practitioner demographics, professional characteristics and self-reported competencies in keratoconus (KC) diagnosis and management. Continuous variables such as age and years of experience were categorised into ordinal groups for analytical consistency. Composite competency scores for KC diagnosis and management were derived from mean ratings of knowledge, skill, and experience, and subsequently dichotomised to generate binary outcomes representing “above” and “below” average competency. Frequencies and percentages were presented for categorical variables. Tables and bar charts were used to illustrate practitioner characteristics, facility distributions and competency profiles. Associations between practitioner characteristics, facility type and competency outcomes were assessed using Fisher’s Exact Test for categorical variables. Multivariable logistic regression models were fitted to identify independent predictors of KC diagnostic and management competency. Adjusted odds ratios (aORs) with 95% confidence intervals (CIs) were reported, with statistical significance defined as *p* < 0.05. Post-estimation analyses in R, including marginal effects and predictive probability plots, were used to visualise adjusted competency probabilities across education levels and age groups.

## 3. Results

A total of 110 eye care practitioners with a mean age of 35.4 years (+/−7.4) and mean professional experience duration of 8.2 (+/−6.2) years responded to the questionnaire, representing a response rate of 58.5% (*n* = 188). Most practitioners were male (70.9%), with 49.1% aged between 30 and 39 years and approximately one-third (31.7%) having at least 10 years of practice experience. The majority held undergraduate (44.6%) or diploma (30.9%) qualifications, while 25.6% had postgraduate training. Over half (52.7%) trained at Mzuzu University, followed by the Malawi College of Health Sciences (34.6%), with the majority now based in the Southern Region (54.6%). Optometrists (60.9%) comprised the largest professional group, with smaller proportions of ophthalmic clinical officers (18.2%), ophthalmologists (6.4%), and cataract surgeons (6.4%). Overall, a significant number of practitioners (57.3%) practiced at a tertiary level of care (Table 1).

### 3.1. Self-Reported Competency Scores for Keratoconus Diagnosis and Management

Figure 1 shows the mean self-reported knowledge, skill, and experience scores (with 95% confidence intervals) for KC diagnosis and management among eye care practitioners. Overall, the diagnostic competency scores were marginally higher than management scores across all indicators. For the diagnosis domain, the mean knowledge, skill and experience scores were 3.60 (95% CI 3.40–3.80), 3.50 (3.30–3.69) and 3.37 (3.18–3.57), respectively, with an overall composite mean of 3.48 (3.30–3.67). For the management domain, the corresponding mean scores were 3.32 (3.10–3.53) for knowledge, 3.23 (3.03–3.42) for skill and 3.21 (3.01–3.40) for experience, yielding an overall mean of 3.25 (3.06–3.44), as presented in Figure 1 below.

### 3.2. Diagnosis and Management Knowledge, Skills, and Experience Across Professions

Ophthalmologists consistently rated themselves higher in both diagnosis and management compared to other professions, reaching the top of the 0–5 scale. Knowledge and skills in diagnosis showed much clearer distinctions between professions than management metrics, where scores were more tightly clustered (with the exception of ophthalmologists). Optometrists typically held the second-highest scores, ranging from 3 to 4, particularly in keratoconus diagnosis knowledge and skills (Figure 2).

### 3.3. Associations Between Practitioner Characteristics and Competency Scores

Significant associations with diagnostic competency were observed for education level (*p* = 0.005), profession (*p* = 0.003), and training institution (*p* = 0.013) (Table 2). Higher diagnostic competency was reported among practitioners with postgraduate qualifications, optometrists and ophthalmologists, and graduates of Mzuzu University or KUHES. For management competency, profession was the only significant correlate (*p* = 0.046), with optometrists and ophthalmologists reporting higher competency than other cadres. No significant associations were observed between competency and sex, age, or years of experience (Table 2).

### 3.4. Factors Associated with Diagnostic Competency for Keratoconus

In the multivariable logistic regression model, education level was significantly associated with diagnostic competency for keratoconus. Practitioners with an undergraduate degree had 3.3 times higher odds of demonstrating diagnostic competency compared to those with a diploma (aOR = 3.3; 95% CI: 1.13, 9.63), while those with a postgraduate qualification had 6.6 times higher odds (aOR = 6.6; 95% CI: 1.91, 22.62). No significant associations were observed for sex or age group after adjustment for other covariates (Table 3).

#### Keratoconus Management Competency by Education

The adjusted probability of demonstrating diagnostic competency increased with higher education level, rising from 0.30 among diploma holders to 0.73 among postgraduates, suggesting a positive association between education level and self-reported diagnostic competency (Figure 3).

### 3.5. Factors Associated with Management Competency for Keratoconus

After adjustment for gender, age and profession, practitioners aged ≥40 years showed a tendency toward higher management competency, although the association did not reach conventional statistical significance after adjustment (AOR = 4.76; 95% CI 0.98–23.02; *p* ≈ 0.05). Although optometrists and ophthalmologists showed significantly higher crude odds of competency (cOR = 5.19 and 8.75, respectively), these associations were attenuated and lost significance after adjustment for covariates. No statistically significant association was observed between gender and management competency (Table 4).

#### KC Management Competency by Age Group

The adjusted probability of demonstrating management competency increased progressively with age, from 0.32 among practitioners under 30 years to 0.69 among those aged 40 years or older (Figure 4).

### 3.6. Current Scopes of Clinical Practice and Service Delivery

Across all six diagnostic instruments (keratometer, slit lamp, retinoscope, tomographer, corneal topographer and pachymeter), usage did not significantly differ by facility type (*p* > 0.05 for all). Conversely, practitioner role in screening for KC varied significantly by facility type (*p* = 0.036), and reported screening activities were predominantly conducted in public hospitals (88%) compared to private (8.1%) and non-governmental organisations (4.1%). Availability of KC management protocols (*p* = 0.012) and co-management of KC prognosis (*p* = 0.003) were also significantly higher in public hospitals (>85%). Additionally, key clinical management roles showed strong associations with facility type, including education (*p* = 0.005), specialised contact lens fitting (*p* = 0.021), basic rigid gas permeable (RGP) fitting (*p* = 0.004) and basic soft contact lens provision (*p* = 0.001). Only spectacle provision did not differ significantly (*p* = 0.091), as shown in Table 5.

### 3.7. Barriers to KC Service Delivery (Diagnosis and Management)

The most common barriers to keratoconus service delivery were lack of equipment, the high cost of contact lenses, and lack of expertise (Figure 5).

#### Barriers by Facility Type, Region and Level of Care

Most reported barriers showed no significant variation by facility type, geographic region, or level of care. However, facility type was significantly associated with reported lack of equipment (*p* = 0.035) (Table 6).

## 4. Discussion

This study provides, to our knowledge, one of the first comprehensive national assessments of eye care practitioners’ competencies, clinical practices, service delivery patterns, and barriers related to keratoconus (KC) management in Malawi. The findings reveal moderate overall competency, stronger confidence in diagnosis than management, significant disparities by educational attainment and professional cadre, and persistent health system constraints that may limit effective KC service delivery.

### 4.1. Practitioner Demographics and Workforce Distribution

The study population was relatively young, with a mean age of 35.4 years and a median of 7 years of professional experience (Table 1). Comparable demographic profiles have been reported in other sub-Saharan African eye care workforce studies, where early-career practitioners constitute a large proportion of providers involved in refractive and anterior segment eye care services [20,21,22]. This trend may be attributable to the relatively recent establishment of ophthalmic training programmes in Malawi, which has resulted in an increasing number of newly qualified practitioners entering the workforce. The predominance of optometrists (60.9%) observed in this study mirrors global trends in which optometrists are increasingly recognised as primary providers of care for corneal conditions, including keratoconus (KC) [23]. Additionally, optometry programmes in Malawi have a higher annual student intake than other eye care training programmes, enrolling approximately 30 students per year. This may explain the greater representation of optometrists within the eye care workforce and their prominent role in the provision of KC-related services.

However, a marked imbalance in workforce distribution was observed (Table 1), with more than half of all eye care practitioners located in the Southern Region (54.6%), particularly within tertiary-level facilities. This pattern is consistent with previous studies demonstrating the concentration of ophthalmic personnel around major referral hospitals, urban centres, and training institutions in Malawi [24,25]. The predominance of practitioners in the Southern Region is likely attributable to the presence of the country’s largest urban centres (Blantyre and Zomba), two central hospitals, and the highest population density. Consequently, a substantial urban–rural disparity exists in the distribution of eye care personnel, potentially limiting access to specialised eye care services for rural populations [26,27]. In a health system already constrained by shortages of skilled personnel, medical supplies, and diagnostic equipment, such maldistribution may further compromise the delivery of keratoconus (KC) services, particularly in underserved areas.

Human resource shortages were also evident when practitioner-to-population ratios were compared with international benchmarks. While developed countries commonly achieve optometrist-to-population ratios of approximately 1:10,000, the World Health Organization (WHO) recommends a minimum ratio of 1:50,000 [28]. Across sub-Saharan Africa, workforce densities remain substantially lower, averaging approximately 2.5 ophthalmologists per million population (1:400,000) and around 10 optometrists per million population (1:100,000) [29]. Based on Malawi’s estimated population of 22.7 million, the findings indicate a considerable deficit in eye care personnel, with one ophthalmologist serving approximately 3.2 million people and one optometrist serving about 338,000 people. Regional disparities were also apparent, with practitioner-to-population ratios ranging from 1:165,521 in the Southern Region to 1:115,262 in the Northern Region, highlighting inequities in workforce distribution and limited availability of eye care services.

Although Malawi’s Ministry of Health has promoted patient satisfaction as part of its broader commitment to Universal Health Coverage (UHC) [30], the substantial shortage and uneven distribution of eye care personnel raise concerns regarding equitable access to services. Efforts to strengthen KC service delivery will require strategic investment in workforce development, recruitment, and retention across all levels of the health system. Expanding the availability of appropriately trained eye care professionals and improving their geographic distribution may enhance access to KC diagnosis and management while supporting broader eye health goals. Lessons from workforce and service delivery models implemented in comparable low-resource settings may also provide valuable guidance for strengthening KC care in Malawi [31].

### 4.2. Self-Reported Competencies in KC Diagnosis and Management

Overall, practitioners reported moderate competency in both keratoconus (KC) diagnosis and management, with diagnostic competency scores consistently exceeding management competency scores across the knowledge, skills, and experience domains (Figure 1 and Figure 2). This disparity suggests that practitioners may be more confident in identifying KC than in providing its ongoing clinical management. Furthermore, the variation in self-reported competency across professional groups indicates differences in training, clinical exposure, and experience, highlighting potential areas for targeted professional development.

Ophthalmologists reported the highest mean scores in both diagnosis and management, with particularly strong confidence in diagnostic competencies. This finding is consistent with studies from other low- and middle-income countries that have demonstrated higher KC-related competency among practitioners with specialist ophthalmic training [31,32,33]. In contrast, optometrists reported lower competency scores in the management domain, particularly within the skills and experience components. These findings may reflect limited exposure to specialised keratoconus interventions, including rigid gas permeable (RGP) lens fitting, scleral lens fitting, and the co-management of patients undergoing corneal collagen cross-linking (CXL).

Similar gaps in KC management competencies have been reported in both high- and low-resource settings, where inadequate access to specialised equipment, training opportunities, and treatment modalities constrains practitioner development [32,33,34,35,36]. Comparable challenges have also been documented in sub-Saharan African countries, including Kenya [37], Mozambique [38], and South Africa [5], where strengthening practitioner training through continuing professional development and curriculum enhancement has been recommended to improve KC care [5,37,38]. Given Malawi’s limited ophthalmology workforce and competing demands on specialist services, reliance on ophthalmologists for the management of all KC cases may not represent the most efficient use of available human resources. Internationally, the majority of non-surgical KC cases are managed using contact-lens-based approaches, which fall within the core scope of optometric practice. Consequently, the management competency gaps identified among optometrists in this study highlight an opportunity to strengthen KC service delivery through targeted upskilling initiatives, particularly in advanced contact lens fitting and co-management of KC patients. Enhancing these competencies may improve access to KC care, promote more efficient utilisation of the eye care workforce, and reduce pressure on specialist ophthalmic service.

### 4.3. Determinants of Diagnostic and Management Competency

Education emerged as a strong predictor of diagnostic competency. Practitioners with postgraduate training had 6.6-fold higher odds of demonstrating diagnostic competency compared to diploma-trained practitioners (Table 3 and Figure 4). This is consistent with evidence that advanced training improves clinical reasoning, interpretation of corneal imaging and diagnostic accuracy for ectatic disorders such as KC [37]. Furthermore, professional discipline was also associated with competency in univariate analysis, with optometrists and ophthalmologists outperforming other groups, more likely due to greater exposure to corneal imaging tools and refractive diagnostics [37,38,39]. However, after adjustment, the effect for KC management competency diminished, suggesting that experience and age may play more meaningful roles in management proficiency (Figure 4). For instance, practitioners aged ≥40 years reported a higher probability of management competency (AOR 4.76), possibly reflecting cumulative clinical exposure and increased involvement in complex case management. Similar age-related competency gradients have been documented in global ophthalmic workforce studies [39,40].

These findings indicate the need for more continuous professional development programmes and/or undergraduate curriculum review to ensure the inclusion of appropriate, updated keratoconus didactic and clinical education. Further, experienced practitioners, reporting skills efficiency within respective facilities, should be encouraged to mentor young practitioners as a strategic priority towards capacity development. Knowledge and skills are paramount in both accurate KC diagnosis and management if the reduction in visual impairment due to this condition is to be realised in Malawi.

### 4.4. Clinical Practice Patterns and Facility-Level Disparities

The findings of this study indicate that keratoconus (KC) diagnostic and management services in Malawi are concentrated within a relatively small number of facilities, particularly public hospitals that possess most of the available diagnostic equipment and are more likely to provide specialised services such as contact lens fitting, rigid gas permeable (RGP) lens dispensing, co-management practices, and established management protocols. While public hospitals are located across both urban and rural settings, specialised KC services appear to be concentrated at higher levels of care, where trained personnel and diagnostic resources are more readily available. This pattern may create challenges for patients who initially present to primary or secondary facilities that have limited capacity for KC diagnosis and management.

The observed concentration of services is likely influenced by broader health system factors, including shortages of specialised eye care personnel, limited availability of diagnostic equipment, and restricted access to advanced treatment options such as specialised contact lenses and corneal collagen cross-linking (CXL). As a result, patients may need referral to facilities with greater diagnostic and management capacity, potentially increasing travel, cost, and waiting-time burdens. Similar service distribution patterns have been reported in other low- and middle-income countries, where KC care is largely provided at secondary and tertiary referral centres [5,7,37,38].

These findings suggest a need to strengthen KC service capacity across multiple levels of the health system. Potential strategies include expanding access to diagnostic equipment, enhancing practitioner training, strengthening referral pathways, and developing nationally coordinated clinical guidelines. In addition, collaboration between public, private, and mission-sector providers may help improve the availability and continuity of KC services while promoting more equitable access to diagnosis and management throughout the country [37,41,42,43].

### 4.5. Barriers to KC Diagnosis and Management

The principal barriers identified in this study were lack of equipment, high cost of contact lenses, and lack of expertise (Figure 5), highlighting challenges at both the health-system and practitioner levels. Although most of these barriers were reported consistently across geographic regions and levels of care, with no statistically significant differences observed (all *p* > 0.05) (Table 6), the prominence of equipment shortages, which was significantly associated with facility type (*p* = 0.035) (Table 6), is unsurprising given the dependence of KC diagnosis on specialised diagnostic technologies. Corneal topographers, tomographers, and pachymeters are critical for the early detection, diagnosis, and monitoring of keratoconus progression; however, their availability remains limited in many low-resource settings [31,37,42,43]. This shortage appears to disproportionately affect public facilities, which often manage the largest burden of keratoconus patients [13,44], potentially constraining timely diagnosis, disease monitoring, and appropriate clinical management. Across all settings, the high cost of contact lenses emerged as the most frequently reported barrier, highlighting affordability as a major challenge to effective KC management. Contact lenses, particularly rigid gas permeable, hybrid, and scleral designs, remain the cornerstone of visual rehabilitation for many KC patients [31,37,43]. Financial barriers may, therefore, limit treatment uptake, reduce adherence, and contribute to avoidable visual impairment.

Although statistical differences were not evident across regions or levels of care, notable trends were observed (Table 6). Equipment shortages and limited clinical expertise were reported more frequently in primary and secondary facilities, reflecting the concentration of specialist personnel and diagnostic resources at tertiary institutions. Consequently, lower-level facilities, which usually serve a majority of rural patients, may face difficulties in early KC screening and detection, and often rely on referrals to tertiary facilities. Other challenges, including weak referral systems, communication gaps, and policy limitations, were relatively uniform across the healthcare system, indicating broader structural weaknesses in service delivery. Collectively, these findings emphasise the need for nationwide interventions focused on improving access to affordable contact lenses, strengthening workforce training, enhancing equipment distribution, and improving referral pathways. While the absence of significant differences suggests that barriers are widely shared, larger studies may be required to further explore potential disparities and guide targeted resource allocation.

## 5. Recommendations

The findings underscore the need for the following:

Strengthened undergraduate, postgraduate and subspecialty training, particularly focusing on keratoconus diagnosis and management that includes corneal imaging, contact lens fitting and CXL.

Redistribution or scaling of workforce deployment to address regional disparities.

Future studies with comparative curriculum evaluations should complement self-reported competency with objective assessments such as standardised clinical examinations (OSCEs), case-based assessments, corneal imaging interpretation tests, direct observation of clinical practice, and clinical audits to determine the extent to which perceived competency corresponds with actual clinical performance.

The development of a national KC Multidisciplinary Management algorithm, as advocated in similar resource-limited contexts [31].

Task-sharing models for KC screening and follow-up, drawing on successful examples from other LMICs [37].

Mobile corneal imaging services using portable topographers and teleconsultation networks linking lower-level facilities with tertiary corneal specialists, regional mentorship programmes, and strategic public–private partnerships to improve access to specialised contact lenses.

## 6. Strengths and Limitations

Additionally, a major strength of this study is that it employed a national census approach, inviting all registered eye care practitioners practicing in Malawi during the study period. Although the final sample comprised 110 participants, representing 58.5% of the eligible national workforce, respondents were drawn from all three regions and from public, mission, and private sectors across primary, secondary, and tertiary levels of care, enhancing the national representativeness of the findings. Nevertheless, non-response bias cannot be completely excluded because not all eligible practitioners participated. Furthermore, the availability of diagnostic equipment, contact lenses and facility resources was based on practitioners’ self-reports and was not independently verified through facility audits or direct observation. Future studies incorporating facility assessments would provide objective confirmation of resource availability.

## 7. Conclusions

This study demonstrates moderate self-reported competency in keratoconus diagnosis and management among eye care practitioners in Malawi, with diagnostic competency generally exceeding management competency. Higher educational attainment was the strongest predictor of diagnostic competency, while ophthalmologists and optometrists consistently reported greater confidence than other professional cadres. Despite the concentration of KC services within public hospitals, major barriers, including inadequate equipment, limited expertise, and the high cost of contact lenses, continue to constrain service delivery. Strengthening the human workforce, KC management policies, specialised training, and access to diagnostic technologies, improving the affordability of contact lens services, and developing standardised management pathways may enhance the quality of keratoconus care and improve visual outcomes in Malawi.

## Figures and Tables

**Figure 1:**
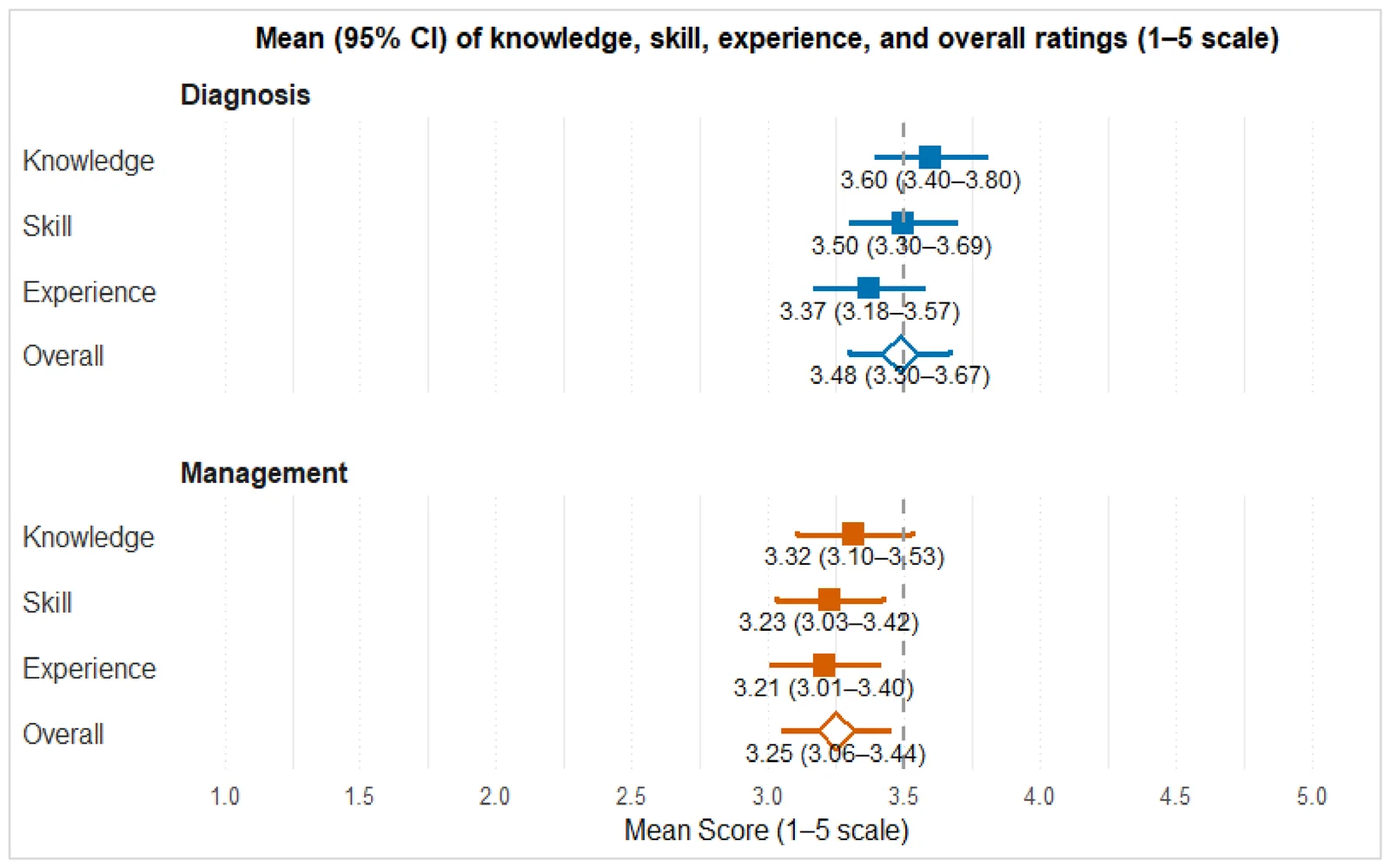
Mean (95% CI) self-reported competency scores for knowledge, skill, experience, and overall competency in keratoconus diagnosis and management among eye care practitioners (5-point Likert scale).

**Figure 2:**
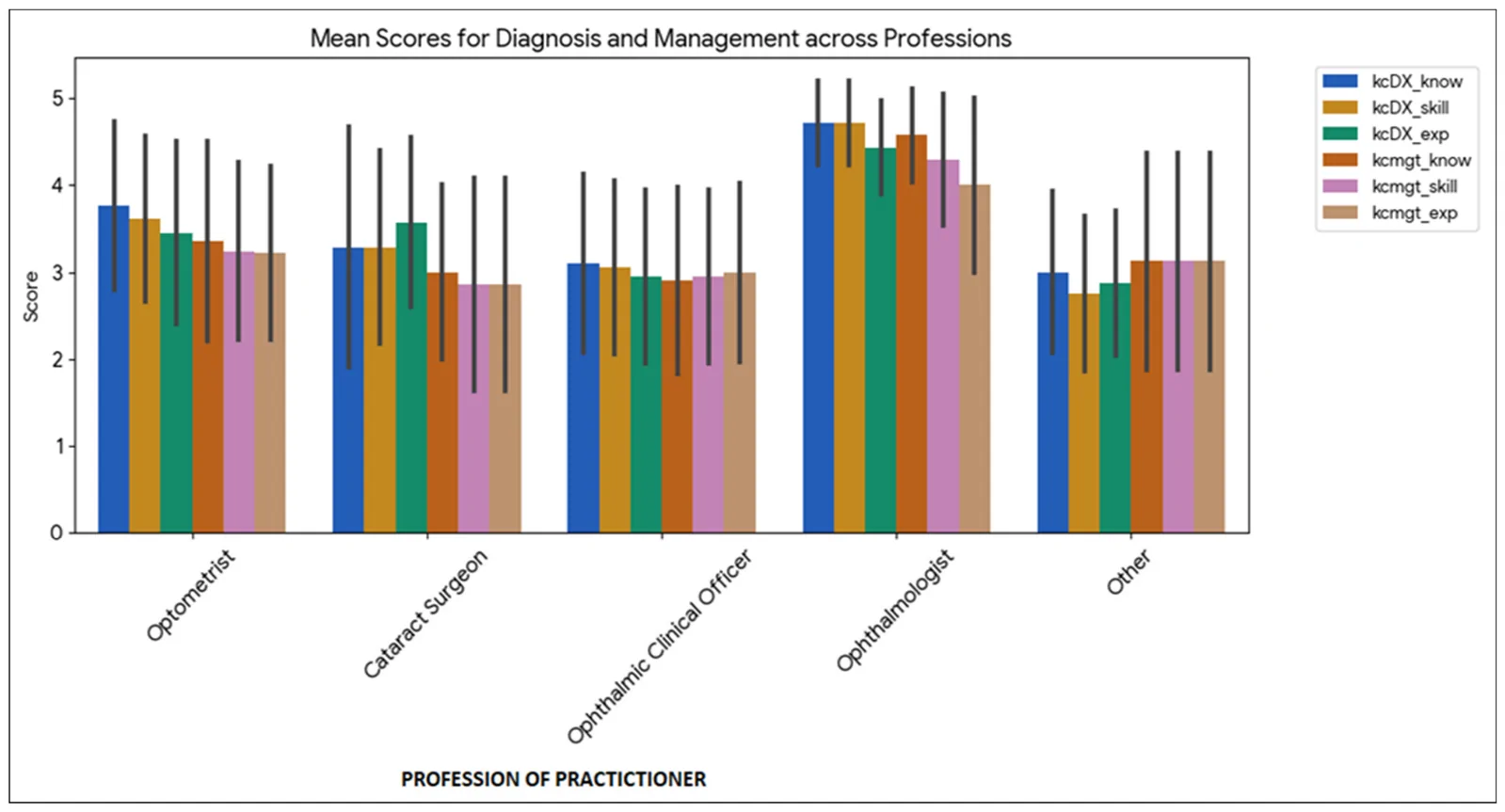
Diagnosis and management knowledge, skills, and experience across professions (*Key: kcDx* = Keratoconus diagnosis, *kcmgt* = Keratoconus management).

**Figure 3:**
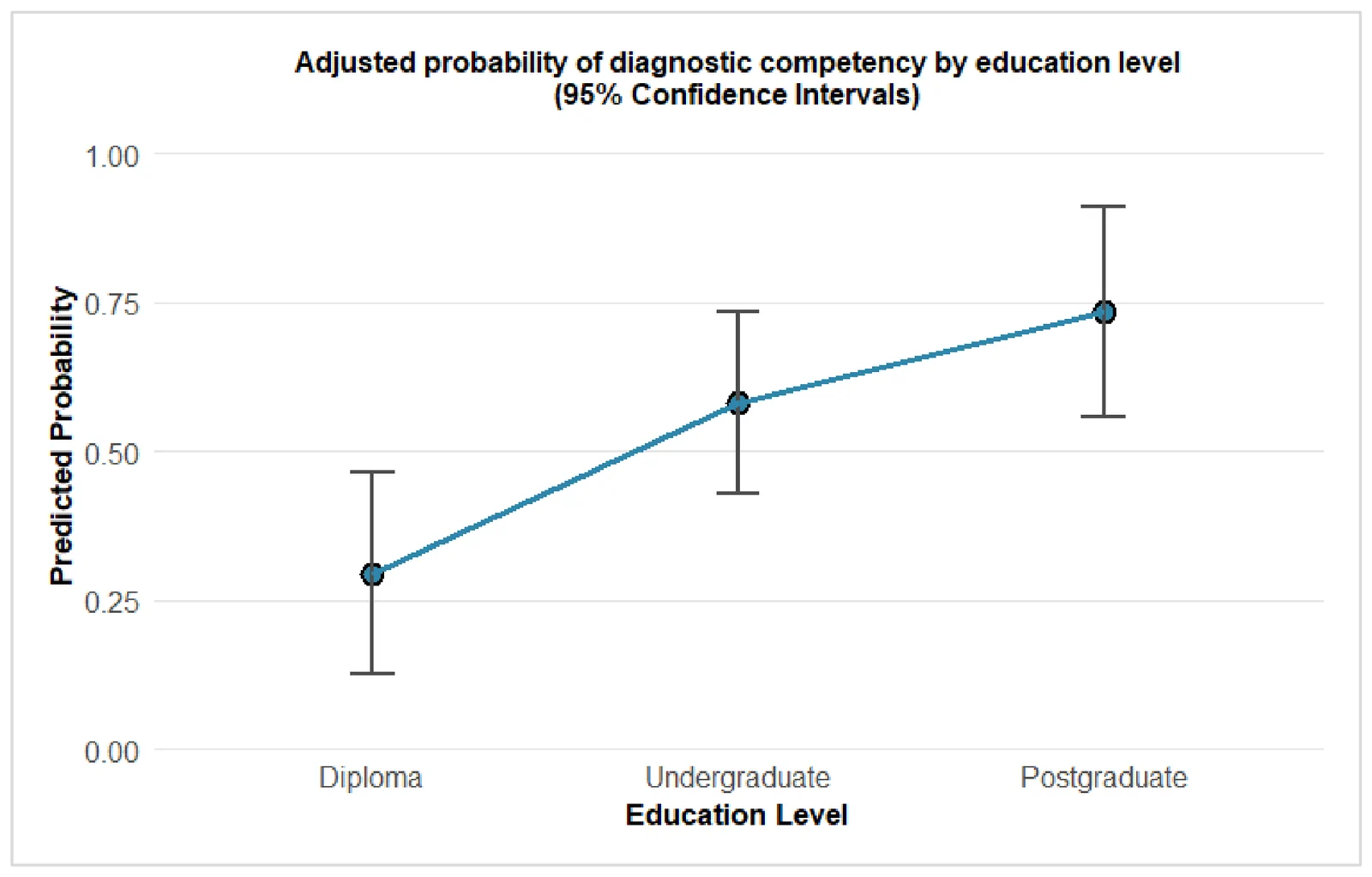
Adjusted predicted probability of diagnostic competency by education.

**Figure 4:**
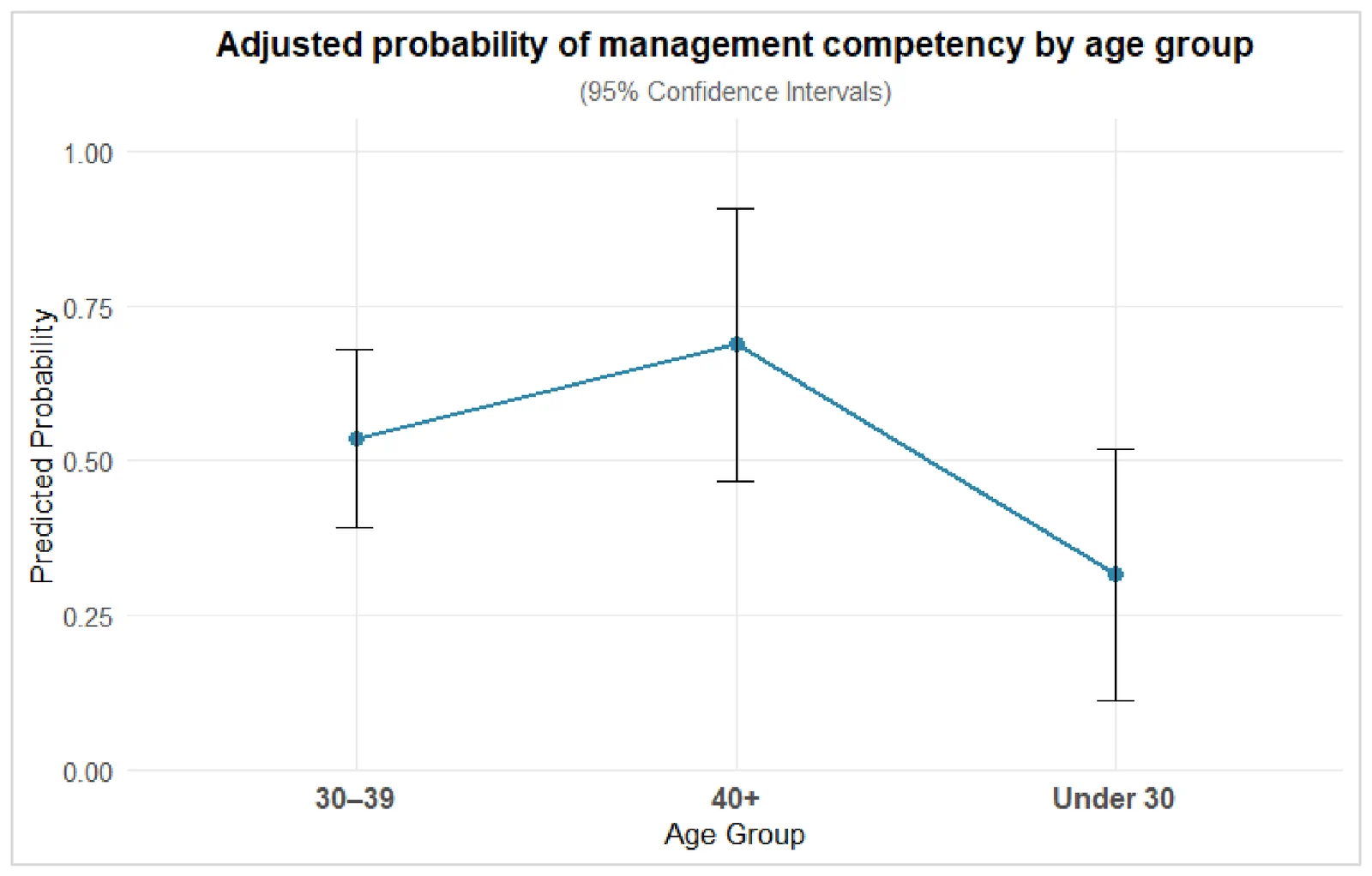
Adjusted predicted probability of KC management competency by age group.

**Figure 5:**
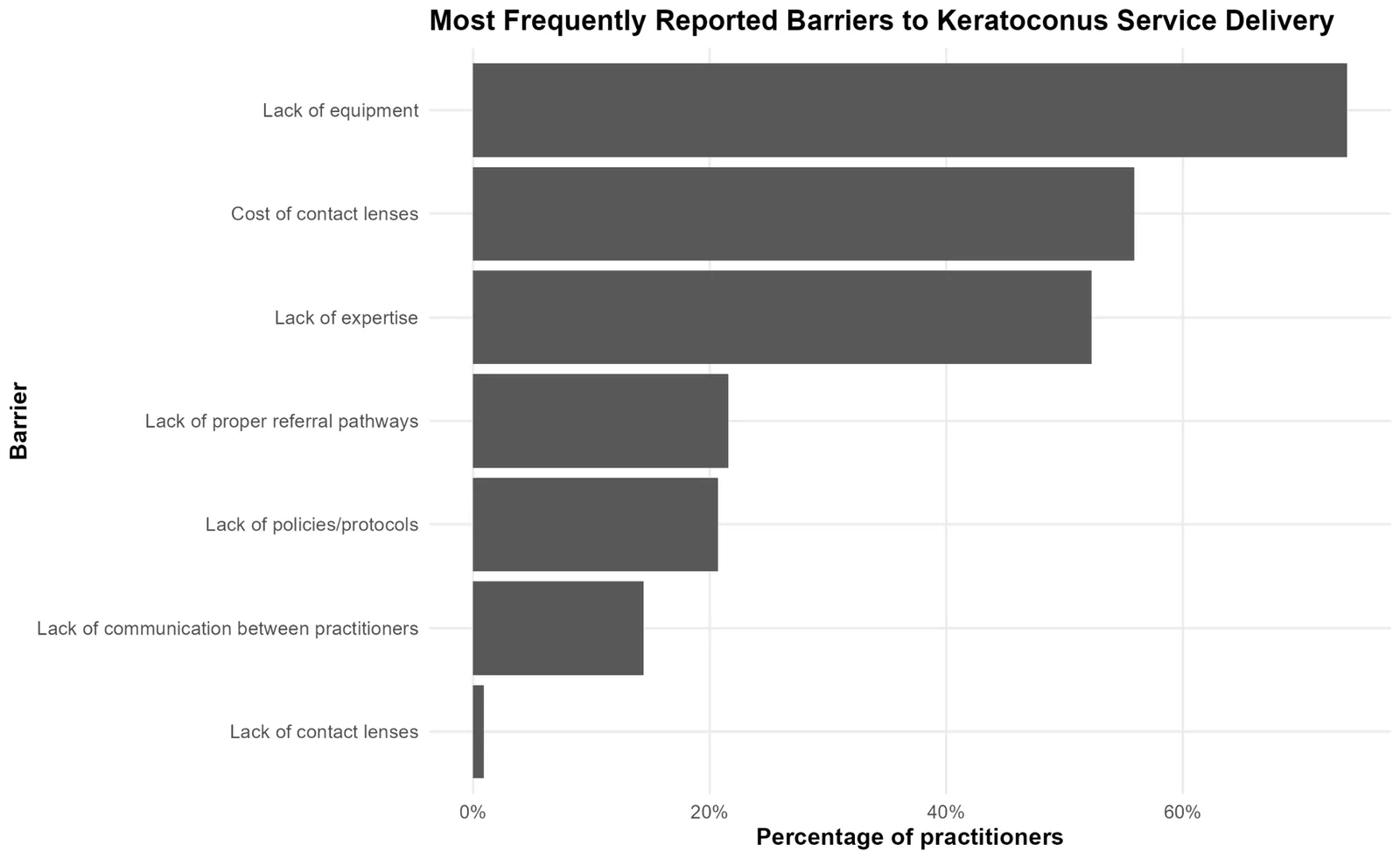
Most frequently reported barriers to KC service delivery by practitioners.

**Table 1:** Demographic and professional workforce characteristics (n = 110).

Characteristic	Obs * (*n* = 110)	Category	N (%)	Mean (SD)
**Age (years)**	106			35.4 (7.4)
**Experience (years)**	101			8.2 (6.2)
Gender	110	Male	78 (70.9)	
		Female	32 (29.1)	
Age	106	<30	26 (24.5)	
		30–39	52 (49.1)	
		40+	28 (26.4)	
Experience (years)	101	<5	35 (34.7)	
		5–9	34 (33.7)	
		10+	32 (31.7)	
Level of Education	110	Postgraduate	27 (25.6)	
		Undergraduate	49 (44.6)	
		Diploma	34 (30.9)	
Training School	110	Malawi CHS	38 (34.6)	
		Mzuzu University	58 (52.7)	
		KUHES	9 (8.2)	
		Other	5 (4.6)	
Profession	110	Optometrist	67 (60.9)	
		Cataract surgeon	7 (6.4)	
		Ophthalmic clinical officers (OCO)	20 (18.2)	
		Ophthalmologist	7 (6.4)	
		Other	9 (8.2)	
Region	108	Central	24 (22.2)	
		Northern	25 (23.2)	
		Southern	59 (54.6)	
Level of Care	110	Primary	20 (18.2)	
		Secondary	24 (21.8)	
		Tertiary	63 (57.3)	
		Other	3 (2.7)	

Obs * = Observations, *SD* = Standard Deviation, *CHS* = College of Health Sciences, *KUHES* = Kamuzu University of Health Sciences.

**Table 2:** Association of practitioner demographics with diagnostic and management competency.

Variable/Category	N	Diagnosis Score		*p*-Value	Management Score		*p*-Value
		≥Average n (%)	<Average n (%)		≥Average n (%)	<Average n (%)	
Overall competency	110	59 (53.6)	51 (46.4)	—	56 (50.9)	54 (49.1)	—
**Gender**	110						
**Male**	78	46 (58.9)	32 (41.0)	0.095	44 (56.4)	34 (43.6)	0.093
**Female**	32	13 (40.6)	19 (59.4)		12 (37.5)	20 (62.5)	
**Age (years)**	106						
**<30**	26	9 (34.6)	17 (65.4)	0.106	9 (34.4)	17 (65.4)	0.111
**30–39**	52	30 (53.6)	22 (44.0)		29 (52.7)	23 (45.1)	
**≥40**	28	17 (60.7)	11 (39.3)		17 (60.7)	11 (39.3)	
**Education level**	110						
**Postgraduate**	27	20 (74.1)	7 (25.9)	0.005	16 (59.3)	11 (40.7)	0.539
**Undergraduate**	49	28 (57.1)	21 (42.9)		25 (51.0)	24 (49.0)	
**Diploma**	34	11 (32.4)	23 (67.7)		15 (44.1)	19 (55.9)	
**Profession**	110						
**Optometrist**	67	40 (59.7)	27 (40.3)	0.003	34 (50.8)	33 (49.3)	0.046
**Cataract surgeon**	7	3 (42.9)	4 (57.1)		3 (42.9)	4 (57.1)	
**Ophthalmic clinical officer**	20	7 (35.0)	13 (65.0)		7 (35.0)	13 (65.0)	
**Ophthalmologist**	7	7 (100.0)	0 (0.0)		7 (100.0)	0 (0.0)	
**Other**	9	2 (22.2)	7 (77.8)		5 (55.6)	4 (44.6)	
**Experience (years)**	101						
**<5**	35	15 (42.9)	20 (57.1)	0.179	16 (45.7)	19 (54.3)	0.679
**5–9.9**	34	22 (64.7)	12 (35.3)		19 (55.9)	15 (44.1)	
**≥10**	32	16 (50.0)	16 (50.0)		17 (53.1)	15 (46.9)	
**Training institution**	110						
**Malawi CHS**	38	14 (36.8)	24 (63.2)	0.013	18 (47.4)	20 (52.6)	0.121
**Mzuzu University**	58	35 (60.3)	23 (39.7)		28 (48.3)	30 (51.7)	
**KUHES**	9	8 (88.9)	1 (11.1)		8 (88.9)	1 (11.1)	
**Other**	5	2 (40.0)	3 (60.0)		2 (40.0)	3 (60.0)	

*Key: CHS* = College of Health Sciences, *KUHES* = Kamuzu University of Health Sciences.

**Table 3:** Multivariable logistic regression for KC diagnosis competency.

Variable/Category	cOR	95% CI	aOR	95% CI
**Sex**				
Female	Ref			
Male	2.1	0.91–4.85	2.2	0.80–6.14
**Age (years)**				
<30	Ref			
30–39	2.6	0.97–6.85	1.50	0.50–4.48
≥40	2.9	0.96–8.84	2.7	0.73–9.90
**Education level**				
Diploma	Ref			
Undergraduate	2.8 *	1.12–6.96	3.3 *	1.13–9.63
Postgraduate	6.0 **	1.95–18.33	6.6 **	1.91–22.62

***Key:*** *cOR* = Crude Odds Ratio; *aOR* = Adjusted Odds Ratio; *CI* = Confidence Interval. * *p* < 0.05; ** *p* < 0.01.

**Table 4:** Multivariable logistic regression for KC management competency.

Variable/Category	cOR	95% CI	aOR	95% CI
**Sex**				
Female	Ref			
Male	2.10	0.91–4.85	1.36	0.49–3.81
**Age (years)**				
<30	Ref			
30–39	2.58	0.97–6.85	2.49	0.85–7.29
≥40	2.92	0.96–8.84	4.76	0.98–23.02
**Profession**				
Other	Ref			
Optometrist	5.19 *	1.00–26.88	0.71	0.17–3.06
Surgeon/Ophthalmologist	8.75 *	1.24–61.68	1.07	0.15–7.74
Ophthalmic C.O	1.88	0.31–11.64	0.20	0.03–1.35

***Key:*** *cOR* = Crude Odds Ratio; *aOR* = Adjusted Odds Ratio; *CI* = Confidence Interval. * *p* < 0.05.

**Table 5:** Characteristics of clinical practice and facility resources for keratoconus management, by health facility type.

Characteristic	n	Private Practice	Public Hospital	Mission/NGO/Other	*p*-Value
Instrument used for KC diagnosis (yes)		n (%)	n (%)	n (%)	
Keratometer	68	5 (7.4)	57 (83.8)	6 (8.8)	0.523
Slit lamp	91	8 (8.8)	76 (83.5)	7 (7.7)	0.542
Retinoscope	70	10 (14.3)	55 (78.6)	5 (7.1)	0.121
Tomographer	15	0 (0)	13 (86.7)	2 (13.3)	0.301
Corneal topographer	37	2 (5.4)	32 (86.5)	3 (8.1)	0.542
Pachymeter	21	2 (9.5)	17 (81.0)	2 (9.5)	0.9
KC diagnostic tests performed by practitioners					
Screening	74	6 (8.1)	65 (87.8)	3 (4.1)	0.036
Keratometry	54	5 (9.3)	44 (81.5)	5 (9.3)	0.938
Refraction	79	10 (12.7)	61 (77.2)	8 (10.1)	0.206
Pachymetry	22	1 (4.6)	19 (86.4)	2 (9.1)	0.808
Tomo/topography	19	0 (0)	17 (89.5)	2 (10.5)	0.231
Slit lamp examination	75	4 (5.3)	65 (86.7)	6 (8.0)	0.067
Criteria for referral					
Initial diagnosis	49	4 (8.2)	42 (85.7)	3 (6.1)	0.183
Progression detected	29	6 (20.7)	21 (72.4)	2 (6.9)	0.183
VA affected	12	0 (0)	11 (91.7)	1 (8.3)	0.183
No time set	16	0 (0)	13 (81.3)	3 (18.8)	0.183
KC protocols/co-management					
KC management protocol available (yes)	79	8 (10.1)	68 (86.1)	3 (3.8)	0.012
KC co-management (yes)	84	9 (10.7)	72 (85.7)	3 (3.6)	0.003
KC management options applied					
Specialised contact lenses (Scleral and hybrid)	12	4 (33.3)	7 (58.3)	1 (8.3)	0.021
Education	88	7 (8.0)	77 (87.5)	4 (4.6)	0.005
Spectacles	84	10 (11.9)	65 (77.4)	9 (10.7)	0.091
RGP lenses	14	3 (21.4)	7 (50.0)	4 (28.6)	0.004
Basic soft contact lenses	23	7 (30.4)	13 (56.5)	3 (13.0)	0.001

*KC* = Keratoconus, *VA* = Visual acuity, *RGP* = Rigid gas permeable.

**Table 6:** Distribution of barriers by facility type, region and level of care.

Barrier	Facility Type					Geographic Region				Level of Care				
	N	Private	Public	NGO/Mission	*p*-Value	Central	Northern	Southern	*p*-Value	Primary	Secondary	Tertiary	Other	*p*-Value
		Yes-n (%)	Yes-n (%)	Yes-n (%)		Yes-n (%)	Yes-n (%)	Yes-n (%)		Yes-n (%)	Yes-n (%)	Yes-n (%)	Yes-n (%)	
Cost of contact lenses	57	5 (8.8)	46 (80.7)	6 (10.5)	0.624	10 (41.7)	14 (56.0)	32 (54.2)	0.521	8 (40.0)	11 (45.8)	36 (57.1)	2 (66.7)	0.486
Lack of expertise	41	6 (14.6)	31 (75.6)	4 (9.8)	0.361	6 (25.0)	8 (32.0)	26 (44.1)	0.222	10 (50.0)	11 (45.8)	19 (30.2)	1 (33.3)	0.318
Lack of policies	13	3 (23.1)	10 (76.9)	0 (0.0)	0.149	0 (0)	4 (16.0)	9 (15.3)	0.121	4 (20.0)	2 (8.3)	7 (11.1)	0 (0)	0.573
Lack of equipment	43	5 (11.6)	31 (72.1)	7 (16.3)	0.035	9 (37.5)	8 (32.0)	24 (38.0)	0.754	7 (35.0)	12 (50.0)	21 (33.3)	3 (100)	0.075
Lack of communication	15	1 (6.7)	12 (80.0)	2 (13.3)	0.746	2 (8.3)	3 (12.0)	9 (15.3)	0.687	2 (10.0)	2 (8.3)	10 (15.9)	1 (33.3)	0.561
Lack of proper referral path	24	4 (16.7)	18 (75.0)	2 (8.3)	0.402	3 (12.5)	5 (20.0)	15 (25.4)	0.420	5 (25.0)	4 (16.7)	14 (22.2)	1 (33.3)	0.866

## Data Availability

The original contributions presented in this study are included in the article. Further inquiries can be directed to the corresponding author.
